# Rapid Synthesis of Hexagonal-Shaped Zn(Al)O-MMO Nanorods for Dye-Sensitized Solar Cell Using Zn/Al-LDH as Precursor

**DOI:** 10.3390/nano12091477

**Published:** 2022-04-27

**Authors:** Ethar Yahya Salih, Asmiet Ramizy, Osamah Aldaghri, Mohd Faizul Mohd Sabri, Nawal Madkhali, Tarfah Alinad, Khalid Hassan Ibnaouf, Mohamed Hassan Eisa

**Affiliations:** 1 College of Medical Sciences Technologies, The University of Mashreq, Baghdad 10021, Iraq; 2 Physics Department, College of Science, University of Anbar, Anbar 00964, Iraq; asmat_hadithi@uoanbar.edu.iq; 3 Department of Physics, College of Sciences, Imam Mohammad Ibn Saud Islamic University (IMSIU), Riyadh 11623, Saudi Arabia; odaghri@imamu.edu.sa (O.A.); namadkhali@imamu.edu.sa (N.M.); tmenad@imamu.edu.sa (T.A.); khiahmed@imamu.edu.sa (K.H.I.); mhsalim@imamu.edu.sa (M.H.E.); 4 NanoMicro Engineering Laboratory, Faculty of Engineering, University of Malaya, Kuala Lumpur 50603, Malaysia; faizul@um.edu.my

**Keywords:** mixed metal oxides nanorods, dye-sensitized solar cell, Zn/Al-layered double hydroxide, fill factor

## Abstract

This study reports a simple new technique for the preparation of novel hexagonal-shaped mixed metal oxides (MMO) nanorods using Zn/Al-layered double hydroxide (LDH) as a precursor for dye-sensitized solar cell (DSSC) application. The effect of the Zn to Al molar ratio demonstrated a sound correlation between the obtained nanorods’ diameter and the fabricated DSSCs efficiency. Additionally, the optical behavior of the fabricated MMO film as well as the absorption enhancement due to the utilized dye are also demonstrated; a cut-off phenomenon at around 376 nm corresponds to the attained hexagonal nanorods. The open-circuit voltage augmented noticeably from 0.6 to 0.64 V alongside an increase in the diameter of nanorods from 64 to 80 nm. The results indicated that an increment in the diameter of the nanorods is desirable due to the enhanced surface area through which a higher amount of dye N719 was loaded ($0.35\;\mathrm{mM}/{\mathrm{cm}}^{2}$). This, in turn, expedited the transport of electrons within the MMO matrix resulting in an advanced short-circuit current. Of the devices fabricated, *ZA-8* exhibited the highest fill factor and efficiency of 0.37% and 0.69%, respectively, because of its boosted short-circuit current and open-circuit voltage.

## 1. Introduction

Dye-sensitized solar cells (DSSCs) are considered a perfect forthcoming alternative for solar energy applications due to their attractive features such as simple preparation process, environmentally friendly, cost-effective, and relatively high conversion efficiency [1,2]. However, the demands for lower cost, more green, and simple preparation techniques of semiconductor electrodes are of crucial importance for higher DSSCs consideration. Recently, mixed metal oxides (MMOs) obtained from layered double hydroxides (LDHs) as precursors are considered promising photo-anode materials for DSSCs because of their low-cost and straightforward synthesis techniques [1,3,4]. LDHs are two-dimensional (2D) class layered anionic clays which consist of binary metallic ions. LDHs have demonstrated a number of interesting properties in which a wide range of prospective applications are attained, for instance, drug delivery, catalyst, photodetectors, and dye-sensitized solar cells [5,6,7,8,9,10]. The thermal treatment of LDHs results in 2D layer collapse and consequently leads to the formation of MMOs structure [11]. Herein, MMOs have attracted considerable interest among researchers for a wide range of applications, such as anode materials, supercapacitors, and photo-catalysts in both ultraviolet and visible light wavelength regions [12,13,14]. In optoelectronic applications, DSSC in particular, MMOs are newly exhibited as promising photo-electrode materials due to their substantially high surface area, sufficient electron injection efficiency, fast photo-responsive behavior as well as similar energy band gap to that of TiO_2_ and ZnO, the most widely-used materials in DSSC applications [15,16,17,18].

A number of reports in the literature report the utilization of MMOs-based Zn/Al-LDHs nanostructures for DSSC applications. Zhang et al. demonstrated the occurrence of hexagonal plate-like MMO particles through which the highest efficiency obtained was 0.0129% [19]. The same research group also investigated the effect of annealing temperatures on MMO plate-like structures for DSSC application, in which a 0.015% conversion efficiency was acquired [20]. Foruzin et al. reported the fabrication of DSSC using “TiO_2_@MMO” with plate-like morphology; a conversion efficiency of 1.5% was attained due to the active role of TiO_2_ in the MMO matrix [21]. Additionally, plate-like MMO particles were synthesized at a maximum conversion efficiency of 0.22%, with respect to the dye employed [22]. Hexagonal sheet-like MMO particles were reported by Xu et al., wherein the highest efficiency obtained was found to be 1.02% [23]. Wang et al. revealed the effect of graphene oxide on plate-like MMO structure for DSSC application with a conversion efficiency of 0.55% [24]. Despite the relatively high conversion efficiency reported in the literature, simple and straightforward photo-electrode materials with hexagonal MMO nanorods morphology were not found.

In this attempt, this study reports a simple technique for the preparation of novel hexagonal-shaped MMO nanorods using Zn/Al-LDH as a precursor. The obtained MMO nanorod layers were used for the fabrication of photo-electrode for DSSC application. Subsequently, a robust correlation between the diameter of the nanorods, Zn to Al molar ratio, and dye loading amount was established.

## 2. Materials and Methods

### 2.1. Synthesis of Photo-Electrode Materials

Hexagonal-shaped Zn(Al)O-MMO nanorods derived from Zn/Al-LDH were synthesized via a combination of co-precipitation and hydrothermal methods. In particular, Zn/Al-LDH precursor was synthesized as a function of the molar ratio of Zn^2+^ to Al^3+^ (*m*), whereby m= 6, 7, and 8. In this attempt, an aqueous solution was prepared, containing *x* M of Zn(NO_3_)_2_·6H_2_O and *y* M of Al(NO_3_)_3_·9H_2_O under an ambient environment and a constant stirring rate of 750 rpm. Essentially, the pH of the growth solution was preserved at 7 throughout the experiment (under the presence of dropwise addition of sodium hydroxide, 1.25 M) for the conservancy of a homogeneous growth matrix. Subsequently, the obtained white precipitate was processed in an autoclave at 65 °C for 8 h to confirm a successful structure growth. Herein, the subsequent solution was later spin-coated on fluorine-doped tin oxide (FTO, Solaronix, Aubonne, Switzerland) substrate to form a 1 cm^2^ layer. The deposition process was repeated for three cycles in which each cycle was subject to an annealing process at 90 °C for 30 min. Phase transformation of the fabricated layers from LDH to MMO was obtained via a thermal treatment process at 350 °C for 1 h with a heating rate of 5 °C/min. Finally, the fabricated layers were denoted as *ZA-m*, where *m* designates the molar ratio.

### 2.2. Fabrication of Photo-Electrode Based DSSC

In this study, the Pt electrode (50 nm) was deposited on the FTO substrate using a sputtering technique on the counter electrode. Concurrently, the fabricated *ZA-m* layers were immersed in a dye solution containing 5 mM of Ruthenizer 535-bisTBA (Solaronix, N719) for 3 h; the resultant layers were used as the photo-electrode. Afterwards, both the photo-electrode and the counter-electrode were assembled together using an adhesive polymer film (Solaronix, 100 μm), which acts as a sealing and separator element. Hereinafter, electrolyte Iodolyte Z50 redox couple: iodide/triiodide (Solaronix) was fully captivated onto the fabricated electrodes via capillarity.

### 2.3. Characterizations

The thermal stability of the Zn/Al-LDH precursor was investigated via thermogravimetric and differential thermal analysis (Mettler Toledo TGA/SBTA851, Columbus, OH, USA) with a heating rate of 5 °C/min. The structural and morphological features of the fabricated layers were, respectively, studied using X-ray diffraction (XRD, Bruker AXS D8, Beijing, China) under CuKα radiation and 40 kV, as well as field emission scanning electron microscopy (FESEM, Hitachi, SU8030, Tokyo, Japan) operated using 1.20 kV accelerating voltage in conjunction with energy dispersive X-ray analysis (EDX). The optical analysis of the fabricated layers was evaluated using UV–vis spectrometry (Shimadzu, UV- 3600, Kyoto, Japan). Finally, the photovoltaic behavior was performed using Keithley 237 SMU (Cleveland, OH, USA) with an output intensity of 100 mW/cm^−2^ under simulated AM 1.5 G sunlight.

## 3. Results and Discussion

**Figure 1:**
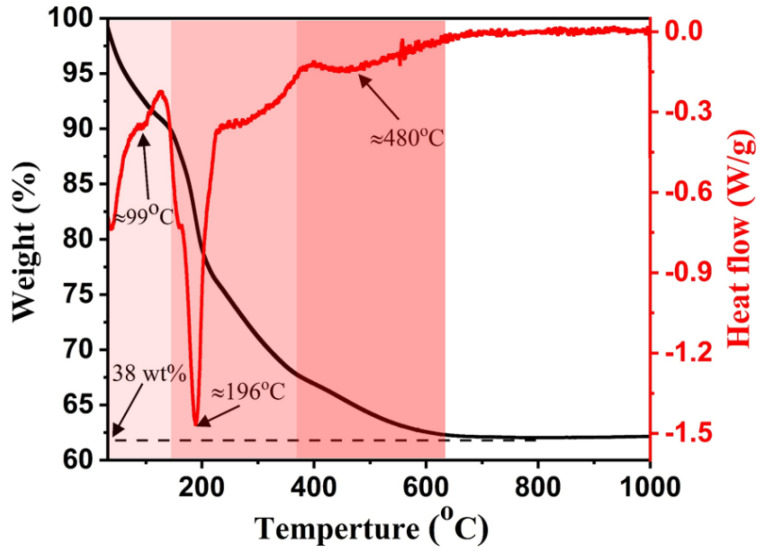
Thermal analysis of pristine LDH with molar ratio of 8:1.

Figure 1 depicts the TGA/DTG analysis of the prepared LDH nanorods with a molar ratio of 8:1, in which three main weight loss stages were attained in the TGA curve; these are due to water evaporation (~37–150 °C), dehydration of brucite-like material in the 2D layers (~150–380 °C), carbonate decomposition, and ZnO formation (~380–620 °C), respectively [25,26]. The addressed weight loss stages exhibited a total of ~38% weight loss. In conjunction, the DTG curve exhibited similar behavior to that obtained in the TGA pattern. Specifically, three foremost DTG peaks can be observed at ~99 °C, 196 °C, and 480 °C due to water release, *ZA-LDH* dehydration, and ZnO recrystallization as well as carbonate decomposition.

**Figure 2:**
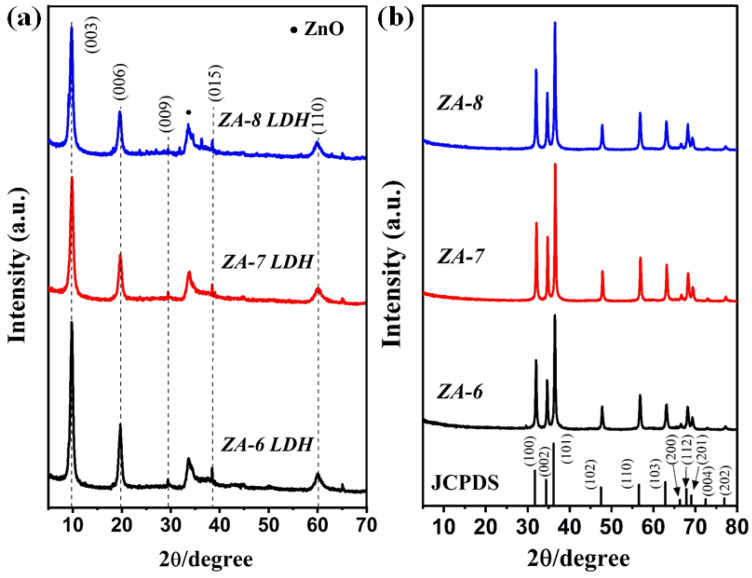
XRD patterns of (**a**) pristine LDH and (**b**) *ZA-m*; where *m* represents the molar ratio.

**Figure 3:**
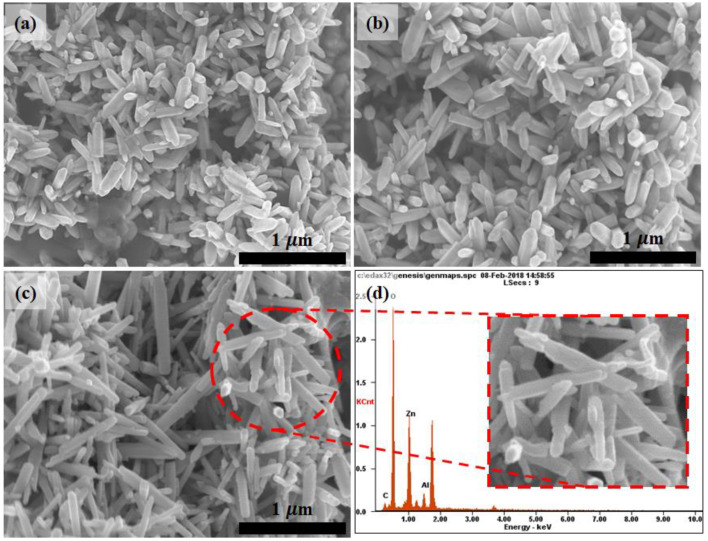
FESEM topographies of the deposited hexagonal MMO nanorods at different molar ratio; (**a**) *ZA-6*, (**b**) *ZA-7*, and (**c**) *ZA-8* and (**d**) EDX spectrum of *ZA-8*.

Figure 2a presents the obtained XRD patterns of the prepared LDH samples wherein four main peaks were noticed corresponding to basal planes (003), (006), and (009) and non-basal planes (110) and (015) similar to the brucite Mg(OH)_2_ structure; this was found to be in accordance with (JCPDS-No:38-0486). Subsequently, the 2D layers structure of LDH collapse, as well as the ZnO crystal formation, can be seen in Figure 2b. A trinary crystal formation was mainly perceived, which can be indexed to the growth of the ZnO structure (JCPDS No: 36-1451). Interestingly, there was no occurrence of Al oxide or Al ions which evidences the substitution of Al^3+^ within the MMO matrix [27]. However, the presence of Al^3+^ in the MMO matrix was confirmed using the EDX technique (Figure 3d). It is generally accepted that the FWHM and crystallite size, obtained using the Debye–Scherer equation can be utilized as crystal quality indicators. Herein, the demonstrated FWHM (Table 1) indicated higher crystal quality at a higher molar ratio. The values obtained for FWHM are 0.309, 0.298, and 0.277 for *ZA-6*, *ZA-7*, and *ZA-8*, respectively.

The top-prospective of the deposited hexagonal *ZA-m*-MMO nanorods at different molar ratios is illustrated in Figure 3. The FESEM indicated that the relative diameter of the hexagonal nanorods alters along with the utilized molar ratio, in which a higher molar ratio resulted in higher average hexagonal-nanorod diameters. In particular, sample *ZA-6* exhibited the lowest diameter value (64 nm), while samples *ZA-7* and *ZA-8* revealed relatively longer hexagonal-nanorods diameters of 75 and 80 nm, respectively. Hence, it is equitable to suggest that there is a direct correlation between the nanorods crystal variation of the deposited MMO and the molar ratio used in this study. The FESEM images also indicate the occurrence of a nanopencil-like morphology, inset in Figure 3c. Additionally, the detection of Al^3+^ within the MMO matrix was confirmed through EDX analysis Figure 3d. The attained FESEM analysis was found to be in an upright agreement with the corresponded XRD analysis, FWHM in particular.

**Figure 4:**
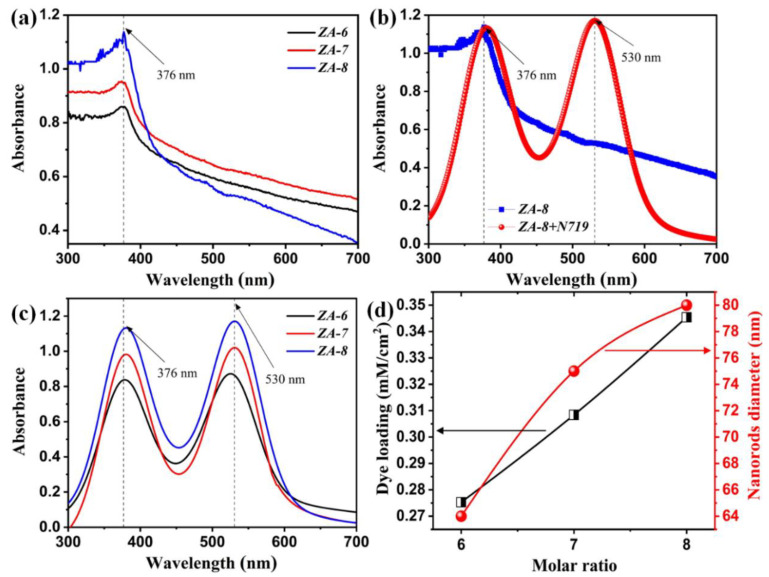
UV-Vis spectra of (**a**) *ZA-m*, (**b**) *ZA-8* and *ZA-8* containing N719, and (**c**) *ZA-m* containing N719, and (**d**) variation of the molar ratio vs. N719 loading and nanorods diameter.

The absorbance spectra of the deposited MMO layers are elucidated in Figure 4a. A cut-off wavelength was noticed at 376 nm, which could be attributed to the attained hexagonal MMO nanorods structure. This phenomenon was observed along with a slight bathochromic shift at a high molar ratio (*ZA-8*), shifting of absorption edge towards a higher wavelength, the so-called red-shift. Continuously, the absorption spectrum of *ZA-8* together with *ZA-8* containing N79 sensitizer is demonstrated in Figure 4b, in which an additional cut-off phenomenon was perceived at 530 nm. The latter is mainly due to N719′s molecules that correspond to the transition between the highest occupied molecular orbital (HOMO) and the lowest unoccupied molecular orbital (LUMO) energy levels; the electromagnetic radiation absorption creates an excited state through electron excitation [28,29]. This, in turn, clearly indicates the essential role of N719 in widening the absorption towards a higher wavelength range. The absorption spectra of the deposited MMO layers containing dye N719 are depicted in Figure 4c, wherein different intensities were obtained. It is suggested, therefore, that the amount of N719 loaded on the MMO layers is directly proportional to the acquired intensities. In conjunction, the amount of N719 loaded was estimated using the UV-Vis technique, wherein the λ max of five N719 concentrations were considered, and a linear fitting equation was created. The resultant linear regression formula (y=4.057x−0.245) was used to calculate the loading amount. Henceforth, a robust relationship between the molar ratio utilized and the amount of N719 loaded was established (Figure 4d). Particularly, the high molar ratio of the deposited MMO resulted in a higher amount of N719 loading on the addressed layer/s. These outcomes were expected from the estimated diameter of the attained hexagonal *ZA-m* nanorods evidencing that a larger nanorod diameter leads to advanced N719 loading (Figure 4d). Such a singularity results in favorable harvesting of light caused by an enlarged surface area and multiple scattering within the nanorods hexagonal structure.

**Figure 5:**
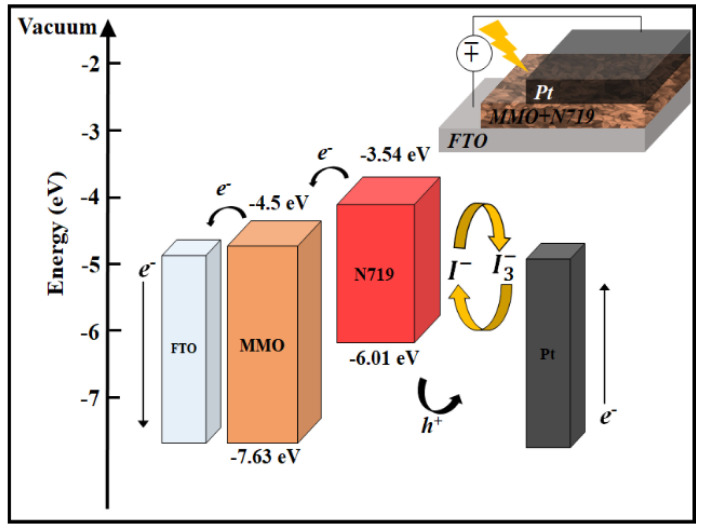
Energy band diagram of MMO and dye N719.

The energy band diagram of the deposited MMO layers and the utilized N719 are presented in Figure 5. Upon illumination, light photons are captured via N719 molecules whereby an electron is excited from HOMO to LUMO in N719, which in turn results in electron injection from the LUMO of N719 into CB of MMO [23]. This, in turn, expedited the transport of electrons within the MMO matrix.

**Figure 6:**
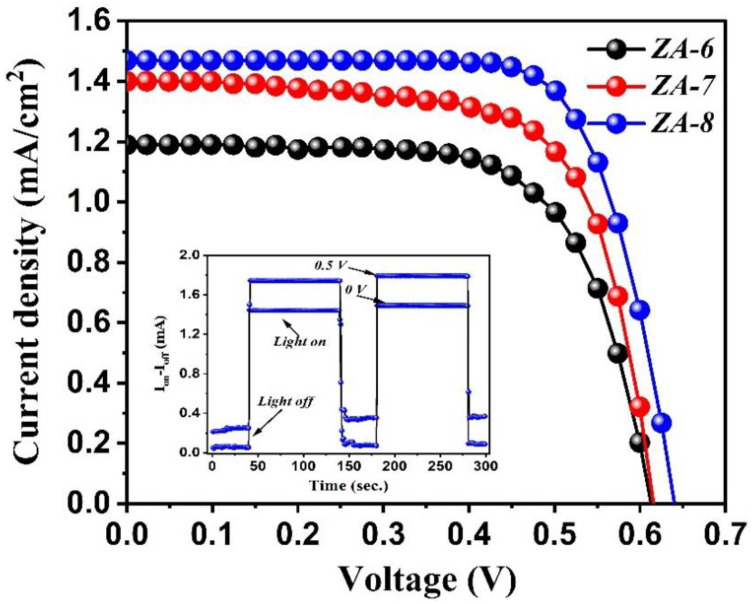
J-V characteristic curves of hexagonal MMO nanorods DSSC.

Continuously, the current density (J)–voltage (V) characteristic curves of the fabricated DSSCs are demonstrated in Figure 6, while the calculated photovoltaic parameters are presented in Table 2. It can be clearly observed that the applied molar ratio has a positive impact on the J-V curve displayed. Specifically, the fabricated DSSC based on *ZA-8* exhibited the highest $J_{sc}$ (1.47), $V_{oc}$ (0.64), fill factor (0.73%), and efficiency (0.69%) as compared to other devices (*ZA-6* and *ZA-7*). DSSCs fabricated based on *ZA-6* and *ZA-7* showed an efficiency of 0.41% and 0.59%, respectively, showing a 20% efficiency enhancement. The demonstrated outcomes agree well with the discussed analysis (FESEM and estimation of dye loading) that a high molar ratio delivers high DSSC performance (Figure 6). Continuously, the stability test of the fabricated *ZA-8* DSSC was confirmed using a switching behavior test for a period of 5 min, through which the addressed device showed a stable behavior at two different applied voltages (inset in Figure 6). Table 3 shows the current study’s maximum J−V characteristics as compared to other reported DSSCs-based Zn/Al-LDH performance; the morphologies reported are demonstrated as well. Our fabricated *ZA-8* DSSCs demonstrated a considerable efficiency (0.69%); however, an efficiency of 1.02% was reported by Xu et al. using the same materials. It should be mentioned that the aforementioned research group proposed a different structure, sheet-like hexagonal MMO particles. Furthermore, Foruzin et al. revealed the occurrence of efficiency as high as 1.5%, which is mainly due to the active role of TiO_2_ within the MMO matrix.

## 4. Conclusions

An easy and new approach for the synthesis of hexagonal MMO nanorods utilizing LDH as a precursor was successfully proposed for DSSC application. The deposited MMO films were characterized using TGA/DTG, XRD, FESEM, and UV-Vis techniques. In particular, the UV-Vis analysis revealed a cut-off phenomenon at 376 nm, which corresponds to the attained hexagonal MMO nanorods. Herein, the effect of the molar ratio between the employed metals (Zn and Al) delivered a pronounced correlation between the microstructural and electrical characteristics of the prepared MMO nanorods and the fabricated DSSCs, respectively. Specifically, increasing the attained MMO nanorods diameter from 64 to 80 nm resulted in higher open-circuit voltage, from 0.6 to 0.64 V, respectively. This was particularly crucial as a larger diameter demonstrated a higher utilized dye N719 loading onto the deposited MMO layers ($0.35\;\mathrm{mM}/{\mathrm{cm}}^{2}$). The fabricated devices, *ZA-8* in particular, demonstrated a considerable fill factor (0.37%) and efficiency (0.69%), which can be attributed to the enhanced short-circuit current and open-circuit voltage.

## Figures and Tables

**Table 1:** In-depth XRD parameters of the prepared Zn(Al)O-MMO Nanorods (hkl=101).

Sample	2θ	FWHM (deg.)	Crystallite Size (nm)
*ZA-6*	36.51	0.309	26.8
*ZA-7*	36.49	0.298	27.8
*ZA-8*	36.58	0.277	29.9

**Table 2:** In-depth XRD parameters of the prepared Zn(Al)O-MMO Nanorods (hkl=101).

Sample	Dye (mM/cm^2^)	Dia. (nm)	$J_{sc}\;\left(\mathrm{mA}\;{\mathrm{cm}}^{-2}\right)$	$V_{oc}$ (V)	FF (%)	η (%)
*ZA-6*	0.28	64	1.17	0.60	0.58	0.41
*ZA-7*	0.31	75	1.40	0.61	0.69	0.59
*ZA-8*	0.35	80	1.47	0.64	0.73	0.69

**Table 3:** J-V characteristics as compared to other studies.

Materials	Structure	$J_{sc}$ (mA ${\mathrm{cm}}^{-2})$	$V_{oc}$ (V)	FF (%)	η (%)	Ref.
Zn/Al-LDH	hexagonal nanorods	1.47	0.64	0.73	0.69	This study
Zn/Al-LDH	hexagonal plate-like	0.073	0.43	0.42	0.013	[19]
TiO2@Zn/Al-LDH	plate-like	2.63	0.81	0.7	4.50	[21]
Zn/Al-LDH	plate-like	1.22	0.49	0.37	0.22	[22]
Zn/Al-LDH	hexagonal sheet-like	2.03	0.69	0.72	1.02	[23]
Zn/Al-LDH	plate-like	4.46	0.37	0.34	0.55	[24]

## Data Availability

The data presented in this study are available on request from the corresponding author. The data are not publicly available due to the fact that a foreseeable project is built on the findings of the current data using a simulation framework.
